# Laparoscopic vs. open anatomical hepatectomy for intrahepatic cholangiocarcinoma: A retrospective cohort study

**DOI:** 10.3389/fsurg.2022.1003948

**Published:** 2022-10-17

**Authors:** Jianlei Wang, Delin Ma, Gang Du, Baokun An, Tong Xia, Tao Zhou, Qingmei Sun, Fengyue Liu, Yadong Wang, Deling Sui, Xiangyu Zhai, Bin Jin

**Affiliations:** ^1^ Department of Organ Transplantation, Qilu Hospital, Cheeloo College of Medicine, Shandong University, Jinan, China; ^2^ Department of Hepatobiliary Surgery, Peking University People's Hospital, Beijing, China; ^3^ Department of Organ Transplantation, Qilu Hospital, Shandong University, Jinan, China; ^4^ Department of Gastroenterology, Qilu Hospital, Shandong University, Jinan, China; ^5^ Department of Anesthesia, Qilu Hospital, Shandong University, Jinan, China; ^6^ Department of General Surgery, The Second People's Hospital of Liaocheng, Liaocheng, China; ^7^ Department of General Surgery, Second Hospital of Shandong University, Jinan, China

**Keywords:** laparoscopic anatomical hepatectomy, open anatomical hepatectomy, outcomes, overall survival, disease-Free survival

## Abstract

**Background:**

Intrahepatic cholangiocarcinoma is a highly malignant and invasive cancer originating from biliary epithelial cells. The current study was designed to evaluate the feasibility, safety, and clinical outcomes of laparoscopic anatomical hepatectomy in patients with intrahepatic cholangiocarcinoma.

**Methods:**

After screening, 95 patients who underwent anatomical hepatectomy for intrahepatic cholangiocarcinoma at our center were enrolled and divided into two groups according to the surgical approach; the baseline characteristics, pathological findings, surgical outcomes, and long-term outcomes were compared. Moreover, univariate and multivariate analyses were performed to identify independent prognostic factors for overall survival (OS) and disease-free survival (DFS).

**Results:**

There were no significant differences in baseline characteristics or pathological findings between the two groups. Regarding short-term outcomes, the intraoperative blood loss, incision length, and length of postoperative hospital stay were more favorable in the laparoscopic anatomical hepatectomy group than the open anatomical hepatectomy group (*P* < 0.05). The two groups differed significantly in the extent of liver resection, with a lower lymph node dissection rate and lymph node yield in the laparoscopic anatomical hepatectomy group (*P* < 0.05). Furthermore, the postoperative complication rate was similar in the two groups (*P* > 0.05). The median postoperative follow-up times were 10.7 and 13.8 months in the laparoscopic anatomical hepatectomy and open anatomical hepatectomy groups, respectively. Regarding the long-term follow-up results, OS and DFS were similar in the two groups (*P* > 0.05). On multivariate analysis, the independent prognostic factors for OS were CA-199, CEA, HGB, tumor diameter, and T stage, and those for DFS were CA-199 (*P* < 0.05), and T stage (*P* < 0.05).

**Conclusion:**

laparoscopic anatomical hepatectomy for intrahepatic cholangiocarcinoma is safe and feasible when performed by experienced surgeons. Compared with open anatomical hepatectomy, laparoscopic anatomical hepatectomy provides better short-term outcomes and a comparable long-term prognosis.

## Introduction

Intrahepatic cholangiocarcinoma (ICC) is a type of cancer originating from biliary epithelial cells, accounting for approximately 5%–10% of primary malignant liver tumors and representing the second most common primary malignant tumor of the liver after hepatocellular carcinoma (1, 2). Moreover, ICC is highly malignant and invasive, with a high relapse rate and poor prognosis. Currently, hepatectomy is considered the primary choice for managing ICC.

Hepatectomy performed in patients with ICC can be divided into anatomical and nonanatomical hepatectomy; anatomical hepatectomy refers to complete resection of the liver segment affected by the tumor on the basis of the Couinaud classification (3). Many studies have found that anatomical hepatectomy is superior to nonanatomical hepatectomy in terms of postoperative survival, complication, and recurrence rates regardless of whether open and laparoscopic surgery is performed (4, 5). Nonetheless, laparoscopic anatomical hepatectomy (LAH) also has several limitations compared with open anatomical hepatectomy (OAH), such as the narrow operating space, and the difficulty in intraoperative bleeding control, all of which bring more challenges to LAH (6). Although there have been many studies comparing LAH with OAH (7), no studies comparing LAH with OAH for ICC have been performed. In addition, ICC is higher invasion, higher recurrence rate and higher mortality compared to hepatocellular carcinoma (HCC), anatomical hepatectomy is more suitable for ICC. To further evaluate the safety and efficacy of LAH for ICC, we retrospectively analyzed 30 patients who underwent LAH and OAH for ICC at our center and compared the short- and long-term outcomes of the patients. In addition, a risk factor analysis was conducted to evaluate the independent prognostic factors for long-term outcomes.

## Materials and methods

### Patients

Between March 2011 and April 2021, a total of 172 consecutive patients underwent hepatectomy for ICC at Qilu Hospital of Shandong University in Jinan (China). The inclusion criteria for our study were as follows: (1) patients who underwent potentially curative resection, defined as complete tumor resection without macroscopic residual tumor tissue, with R0 or R1 surgical margins, and without evidence of distant metastases; (2) patients with ICC confirmed by postoperative pathology; (3) patients who underwent anatomical liver resection; and (4) patients with complete clinical information available. While the exclusion criteria were (1) patients did not undergo radical resection or anatomical resection; (2) The pathological type was not ICC; (3) Incomplete clinical data. After screening, 95 patients who underwent anatomical hepatectomy were finally included in our study. These patients were divided into the LAH group (*n* = 30) and OAH group (*n* = 65) according to the surgical procedure. All of the data used in this study were obtained from our hospital database and anonymized during the study. This study complied with the Declaration of Helsinki (World Medical Assembly) and its amendments and was approved by the Ethics Committee of Qilu Hospital, Shandong University (approval number: KYLL-202011-180).

### Preoperative preparation

Preoperatively, the patients were given the necessary supportive therapy, such as liver protection therapy or oral antiviral therapy, to improve their liver function reserve. All patients underwent contrast-enhanced computed tomography (CT) or magnetic resonance imaging (MRI) before surgery to assess tumor characteristics (morphology, size, number, and location) and to provide guidance for the surgical plan, as well as for the assessment of the patient's residual liver volume.

### Surgical technique

All patients were placed in the supine position after general anesthesia was administered. Routine surgical disinfection and draping were performed. For LAH patients, pneumoperitoneum was routinely established before the surgical procedures, and the pressure was maintained between 12 mmHg and 15 mmHg. OAH was performed through an inverted L-shaped incision in the upper left abdomen, measuring approximately 20–25 cm.

**Figure 1:**
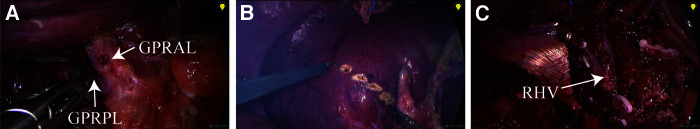
Surgical procedure for laparoscopic anatomical right posterior lobe resection (using the extrahepatic glissonean approach). (**A**) The Glissonean pedicle of the right posterior lobe (GPRPL) was identified, dissected free and then occluded with a bulldog clamp. (**B**) The extent of resection was determined according to the ischemic line, and an electrocoagulation hook was used to draw a pretransected line. (**C**) The right posterior lobe of the liver was removed, and the right hepatic vein trunk was preserved. GPRAL, Glissonean pedicle of right anterior lobe; GPRPL, Glissonean pedicle of right posterior lobe; RHV, right hepatic vein.

**Figure 2:**
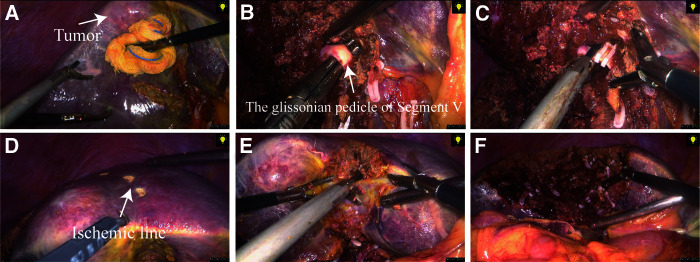
Surgical procedure for laparoscopic anatomical segment V resection (using the hepatic parenchymal transection-first approach). (**A**) Anatomical landmarks or intraoperative ultrasound were used to define the borders of segment V, and a pretransected line was created through ultrasonic dissection. (**B**) The Glissonean pedicle of segment V was dissected free during transection of the liver parenchyma. (**C**) The Glissonean pedicle was clamped and then cut off. (**D**) After inducing ischemia in segment V, we used an electrocoagulation hook to draw a pretransected line based on the ischemic line. (**E**) and (**F**) Complete transection of the remaining liver parenchyma was continued and finally, resection of segment V was completed.

The surgeon performed LAH and OAH following the same standardized surgical principles. After entering the abdominal cavity, the abdominal organs were examined to exclude abdominal metastases, and then the tumor location was assessed to determine the surgical plan. Tumors in superficial locations could be judged by the naked eye, while tumors in deeper locations could be judged by intraoperative ultrasound and other equipment. Subsequently, the liver to be resected was fully mobilized by releasing its surrounding ligaments according to the surgical plan. Before liver resection was performed, a tourniquet was routinely prepared for the Pringle maneuver and was used intermittently to keep the operative field dry when necessary. For procedures involving more extensive liver resection, such as trisectionectomy, hemihepatectomy, central bisectionectomy, and sectionectomy, we preferred the extrahepatic Glissonean approach (Figure 1), which requires the operator to predissect the Glisson system of the hemihepatic or hepatic lobe at the first hepatic portal, subsequently ligating them with vascular clips or silk wires and then severing the liver parenchyma along the ischemic line of the liver surface using an ultrasonic knife to remove the hemihepatic or hepatic lobe. In contrast, for minor liver resection, such as segmentectomy, it is difficult to use the extrahepatic Glissonean approach because the Glisson system of the hepatic segment is located deeper in the hepatic parenchyma in the hilar region. For this reason, we preferred the hepatic parenchymal transection-first approach (Figure 2), in which the surgeon predetermines the peripheral boundaries of the liver segments to be surgically removed based on anatomical landmarks or with the help of intraoperative ultrasound; then, the liver parenchyma is cut first, revealing the Glisson system of the hepatic segment during dissection, which is ligated and cut. Notably, the choice between the above two approaches is not absolute, and these approaches should be used flexibly according to the actual situation encountered intraoperatively. Lymph node dissection (LND) in the hilar region was not routinely performed in all cases at our center; instead, this was performed only in cases in which enlarged lymph nodes were found by preoperative imaging or intraoperative observation.

### Postoperative management

After surgery, all patients fasted and received intravenous nutritional support until gastrointestinal function was restored. Postoperative laboratory tests, such as the complete blood count, biochemical profile, coagulation tests, and liver function tests, were performed every two days during the postoperative recovery course. In addition, CT examination was routinely performed on the fourth postoperative day to assess the patient's intra-abdominal condition.

### Data collection and definitions

We retrospectively collected data from the patient's medical records, including clinical baseline data, laboratory test results, pathological findings, intraoperative data, postoperative data, and follow-up data. Postoperative follow-up was performed once every three months by telephone. Overall survival (OS) was defined as the time from surgery until death, and recurrence-free survival (RFS) was defined as the survival duration without ICC recurrence. We used the Brisbane 2,000 classification to define the anatomical resection procedures (8). The 8th edition of the AJCC/UICC TNM staging system was applied, and perioperative complications were evaluated with the Clavien–Dindo complication classification system (9, 10).

### Statistical analysis

Continuous data are presented as the mean ± standard deviation (SD) or as the median with interquartile range [median (Q1, Q3)] according to their distribution, and Student's *t* test or the Mann–Whitney U test was used for comparisons. Categorical data are presented as numbers with percentages (%) and were compared using the *χ*2 test or Fisher's exact test. OS and disease-free survival (DFS) curves were plotted following the Kaplan–Meier method, and the log-rank test was used to compare the curves. Univariate Cox regression analysis was applied to evaluate the potential risk factors for prognosis; the clinical parameters with *P* < 0.10 were entered into multiple Cox regression analysis to identify independent prognostic factors for OS or DFS. In all analyses, *P* < 0.05 was considered statistically significant. All statistical analyses were performed using SPSS Statistics V.25 (IBM SPSS Software) and/or R V.3.5.3.

## Results

### Preoperative situation

A comparison of the baseline characteristics between the OAH and LAH groups is summarized in Table 1. There were 37 males and 28 females in the OAH group, with an average age of 61.7 years, while the LAH group consisted of 11 males and 19 females, with an average age of 60.6 years; no significant differences were observed between the two groups (*P* > 0.05). There was no significant difference in body mass index (BMI) between the two groups (23.6 kg/m^2^ vs. 24.5 kg/m^2^, *P* = 0.292). There were also no significant differences between the two groups in sex, American Society of Anesthesiology (ASA) score, comorbidities, history of smoking, history of alcohol consumption, history of abdominal surgery, hepatitis B virus infection or intrahepatic biliary lithiasis (*P* > 0.05). The preoperative laboratory test results for tumor markers, such as CA-199, CEA, and AFP, were not significantly different between the two groups (*P* > 0.05); additionally, no significant differences were observed in the other laboratory test results (*P* > 0.05).

**Table 1:** Comparison of the baseline characteristics between OAH and LAH groups.

Variables	OAH group (*n* = 65)	LAH group (*n* = 30)	*P* value
Age, year	61.7 ± 9.0	60.6 ± 9.4	0.616
Sex, *n* (%)			
Male	37 (56.9%)	11 (36.7.0%)	0.066
Female	28 (43.1%)	19 (63.3%)	
BMI, kg/m^2^	23.6 ± 3.6	24.5 ± 3.7	0.292
ASA score, *n* (%)			
1	4 (6.2%)	4 (13.3%)	0.489^a^
2	56 (86.2%)	24 (80.0%)	
3	5 (7.7%)	2 (6.7%)	
Comorbidity, *n* (%)			
Diabetes	7 (10.8%)	3 (10.0%)	1.000^a^
Hypertension	19 (29.2%)	10 (33.3%)	0.686
Coronary heart disease	2 (3.1%)	3 (10.0%)	0.322^a^
History of smoking, *n* (%)	17 (26.2%)	10 (33.3%)	0.471
History of alcohol consumption, *n* (%)	21 (32.3%)	7 (23.3%)	0.373
Previous abdominal surgery, *n* (%)	15 (23.1%)	6 (20.0%)	0.737
Hepatitis B virus infection, *n* (%)	10 (15.4%)	3 (10.0%)	0.749^a^
Intrahepatic biliary lithiasis, *n* (%)	6 (9.2%)	5 (16.7%)	0.315^a^
Laboratory tests			
CA-199, U/ml	163.0 (16.9, 800.0)	109.6 (30.6, 1000)	0.936
AFP, U/ml	3.2 (2.1, 6.0)	3.4 (2.1, 5.6)	0.496
CEA, U/ml	4.0 (2.1, 27.4)	3.2 (2.0, 7.8)	0.223
Neutrophil count, 10^^^9/ml	4.5 ± 2.0	4.3 ± 1.7	0.551
Lymphocyte count, 10^^^9/ml	1.5 (1.3, 1.9)	1.7 (1.3, 2.2)	0.267
Platelet count, 10^^^9/ml	239.6 ± 88.7	241.1 ± 77.3	0.936
HGB, g/L	133.6 ± 16.3	137.5 ± 19.2	0.304
ALT, U/L	23.0 (15.0, 43.0)	19.5 (13.0, 36.0)	0.391
AST, U/L	25.0 (18.0, 39.0)	22.5 (18.0, 34.0)	0.446
TBIL, umol/L	12.8 (8.5, 27.0)	12.6 (10.3, 14.7)	0.428
ALB, g/L	41.7 ± 4.6	43.3 ± 4.0	0.084

LAH, laparoscopic anatomical hepatectomy; OAH, open anatomical hepatectomy; BMI, body mass index; ASA, American Society of Anesthesiology; CA-199, cancer antigen 19-9; AFP, alpha fetoprotein; CEA, carcinoembryonic antigen; HGB, hemoglobin; ALT, alanine aminotransferase; AST, aspartate aminotransferase; TBIL, total bilirubin; ALB, albumin. Data are presented as the mean with standard deviation (x¯ ± SD), or median with interquartile range [median (Q1, Q3)], or counts with percentages *n* (x%).

^a^ Indicates using Fisher exact test.

### Pathological findings

Table 2 shows a comparison of the pathological findings between the OAH and LAH groups. The tumor diameter was clearly larger in the OAH group than in the LAH group (4.7 cm vs. 5.7 cm), but the difference was not statistically significant (*P* = 0.053). In addition, no other significant differences were found between the two groups with respect to other pathological findings, such as tumor number, pathological differentiation, TNM stage, T stage, microscopic metastatic foci, microscopic perineural invasion and microscopic microvascular invasion (*P* > 0.05).

**Table 2:** Comparison of the pathologic findings between OAH and LAH groups.

Variables	OAH group (*n* = 65)	LAH group (*n* = 30)	*P* value
Tumor diameter, cm	5.7 ± 0.3	4.7 ± 0.4	0.053
Tumor number, n (%)			
Single	55 (84.6%)	26 (86.7%)	1.000^a^
Multiple	10 (15.4%)	4 (13.3%)	
Pathological differentiation, *n* (%)			
Poorly differentiated	14 (21.5%)	5 (16.7%)	0.312^a^
Moderately differentiated	46 (70.8%)	25 (83.3%)	
Well differentiated	5 (7.7%)	0 (0.0%)	
TNM stage, *n* (%)			
* *0/IA/IB/II	38 (58.5%)	19 (63.3%)	0.652
* *IIIA/IIIB/IV	27 (41.5%)	11 (36.7%)	
T stage, *n* (%)			
Tis/T1a/T1b/T2	50 (58.5%)	19 (63.3%)	0.291
T3/T4	27 (41.5%)	11 (36.7%)	
Microscopic metastatic foci, *n* (%)	7 (10.8%)	6 (20.0%)	0.335^a^
Microscopic perineural invasion, *n* (%)	16 (24.6%)	9 (30.0%)	0.580
Microscopic microvascular invasion, *n* (%)	13 (20.0%)	7 (23.3%)	0.711

LAH, laparoscopic anatomical hepatectomy; OAH, open anatomical hepatectomy. Data are presented as the mean with standard deviation (¯x ± SD), or counts with percentages *n* (x%).

^a^ indicates using Fisher exact test. Bold indicates statistical significance.

### Surgical outcomes

A comparison of the surgical outcomes between the OAH and LAH groups is shown in Table 3. The operative duration was similar in the two groups (225.3 min vs. 231.0 min, *P* = 0.787). Regarding the type of liver resection, the proportions of trisectionectomy and hemihepatectomy were higher in the OAH group than in the LAH group (3.1% vs. 0.0%, 81.5% vs. 56.7%), while the proportions of central bisectionectomy, sectionectomy, and segmentectomy were lower in the OAH group (4.6% vs. 6.7%, 4.6% vs. 30.0%, 6.2% vs. 6.7%); these differences were statistically significant (*P* = 0.007). In the LAH group, six (20.0%) patients underwent conversion to open surgery. As expected, the incision length was significantly longer in the OAH group than in the LAH group (21.1 cm vs. 11.5 cm, *P* < 0.001). Although the volume of intraoperative blood loss was significantly higher in the OAH group than in the LAH group (300.0 ml vs. 200.0 ml, *P* = 0.044), the rate of intraoperative transfusion did not differ significantly between the two groups (18.5% vs. 10.0%, *P* = 0.375). LND was performed in 37 cases (56.9%) in the OAH group and only 6 cases (20.0%) in the LAH group, and this difference was statistically significant (*P* = 0.001). Among those who underwent LND, in the OAH group, 17 (45.9%) and 20 (54.1%) patients were found to have positive and negative lymph nodes, respectively, while in the LAH group, 1 (16.7%) and 5 (83.3%) patients were found to have positive and negative lymph nodes, respectively. In addition, the patients in the OAH group were more likely to have an adequate lymph node evaluation (lymph node yield ≥6) than the patients in the LAH group (10.8% vs. 0.0%, *P* = 0.001). There were no significant differences regarding the Pringle maneuver, surgical margin, or postoperative transfusion between the two groups (*P* > 0.05). The incidence of severe complications, which were defined as those with a Clavien–Dindo grade ≥3, was higher in the OAH group than in the LAH group, but there was no significant difference (12.3% vs. 3.3%, *P* > 0.05). Furthermore, there were no significant differences between the two groups in terms of specific complications, such as incision-related complications, postoperative complications, delayed gastric emptying, bile leakage, peritoneal effusion, intraperitoneal infection, pleural effusion, lung infection, myocardial infarction, and heart failure (*P* > 0.05). One patient (1.5%) in the OAH group was transferred to the intensive care unit (ICU) because of severe pulmonary infection, while no (0.0%) patients in the LAH group were transferred to the ICU (*P* = 1.000). The mean length of postoperative hospital stay was significantly longer in the OAH group than the LAH group (10.6 days vs. 8.8 days, *P* = 0.031). However, the hospital costs were similar in the two groups (73597.1 RMB vs. 75031.7 RMB, *P* = 0.819), and no deaths within 30 days after surgery were reported in either group.

**Table 3:** Comparison of the surgical outcomes and follow-up outcomes between OAH and LAH groups.

Variables	OAH group (*n* = 65)	LAH group (*n* = 30)	*P* value
Operation *time*, min	225.3 ± 75.4	231.0 ± 103.2	0.787
Intraoperative blood loss, ml	300.0 (170.0, 275.0)	200.0 (100.0, 300.0)	**0** **.** **044**
Intraoperative transfusion, *n* (%)	12 (18.5%)	3 (10.0%)	0.375
Liver resection*, n* (%)			**0**.**007**^b^
Trisectionectomy	2 (3.1%)	0 (0.0%)	
Right-trisectionectomy	1 (1.5%)	0 (0.0%)	
Left-trisectionectomy	1 (1.5%)	0 (0.0%)	
Hemi-hepatectomy	53 (81.5%)	17 (56.7%)	
Right hemi-hepatectomy	14 (21.5%)	5 (16.7%)	
Left hemi-hepatectomy	39 (60.0%)	12 (40.0%)	
Central bisectionectomy	3 (4.6%)	2 (6.7%)	
Sectionectomy	3 (4.6%)	9 (30.0%)	
Left lateral sectionectomy,	1 (1.5%)	8 (26.7%)	
Right posterior sectionectomy	2 (3.1%)	1 (3.3%)	
Segmentectomy	4 (6.2%)	2 (6.7%)	
Conversion, *n* (%)	-	6 (20.0%)	-
Incision length, cm	21.1 ± 2.4	11.5 ± 5.3	**<0**.**001**
Intraoperative transfusions, *n* (%)	12 (18.5%)	3 (10.0%)	0.375^b^
Lymph node dissection, *n* (%)	37 (56.9%)	6 (20.0%)	**0**.**001**
Nodal status			
Positive	17 (45.9%)	1 (16.7%)	** **
Negative	20 (54.1%)	5 (83.3%)	** **
Lymph node yield			
0 nodes	28 (43.1%)	24 (80.0%)	**0**.**001**^b^
1–5 nodes	30 (46.2%)	6 (20.0%)	** **
≥6 nodes	7 (10.8%)	0 (0.0%)	** **
Pringle maneuver, *n* (%)	18 (27.7%)	10 (33.3%)	0.575
*Single*	15 (23.1%)	4 (13.3%)	
*Multiple*	3 (4.6%)	6 (20.0%)	
Surgical margin, *n* (%)			
R0	62 (95.4%)	29 (96.7%)	1.000^b^
R1	3 (4.6%)	1 (3.3%)	
Postoperative transfusion, *n* (%)	2 (3.1%)	3 (10.0%)	0.322^b^
Morbidity of complications, *n* (%)			
Clavien-Dindo ≥3	8 (12.3%)	1 (3.3%)	0.264^b^
Incision-related complications	3 (4.6%)	0 (0.0%)	0.549^b^
Postoperative haemorrhage	2 (3.1%)	1 (3.3%)	1.000^b^
Delayed gastric emptying	1 (1.5%)	0 (0.0%)	1.000^b^
Bile leakage	4 (6.2%)	1 (3.3%)	1.000^b^
Peritoneal effusion	9 (13.8%)	2 (6.7%)	0.493^b^
Intraperitoneal infection	4 (6.2%)	0 (0.0%)	0.304^b^
Pleural effusion	24 (36.9%)	9 (30.0%)	0.510
Lung infection	13 (20.0%)	1 (3.3%)	0.058^b^
Myocardial infarction	1 (1.5%)	1 (3.3%)	0.534^b^
Heart failure	3 (4.6%)	3 (10.0%)	0.376^b^
ICU admission, *n* (%)	1 (1.5%)	0 (0.0%)	1.000^b^
Postoperative hospital stays, days	10.6 ± 3.9	8.8 ± 3.3	**0**.**031**
Hospital cost, RMB	73597.1 ± 31001.7	75031.7 ± 21533.7	0.819
30-day death, *n* (%)	0 (0.0%)	0 (0.0%)	-
Follow-up outcomes			
Subsequent therapy, *n* (%)	16 (24.6%)	7 (23.3%)	0.892
Transarterial liver chemoembolization	6 (9.2%)	6 (20.0%)	0.186^b^
Radiofrequency ablation	4 (6.2%)	2 (6.7%)	1.000^b^
Targeted therapy	3 (4.6%)	1 (3.3%)	1.000^b^
Immunotherapy	1 (1.5%)	0 (0.0%)	1.000^b^
Reoperation	1 (1.5%)	0 (0.0%)	1.000^b^
Chemotherapy	5 (7.7%)	0 (0.0%)	0.176^b^
Radiotherapy	1 (1.1%)	0 (0.0%)	1.000^b^
Follow-up time, months^a^	13.8 (1.1, 72.2)	10.7 (1.0, 66.0)	0.731
Total disease recurrence, *n* (%)	29 (44.6%)	10 (33.3%)	0.299
Total death, *n* (%)	25 (38.5%)	9 (30.0%)	0.424

LAH, laparoscopic anatomical hepatectomy; OAH, open anatomical hepatectomy; RMB, Ren Min Bi. Data are presented as the mean with standard deviation (x ± SD), or median with interquartile range [median (Q1, Q3)], or counts with percentages *n* (x%).

^a^ indicates using Fisher exact test. Bold indicates statistical significance.

^b^ Data are presented as the median with range.

### Follow-up and long-term outcomes

**Figure 3:**
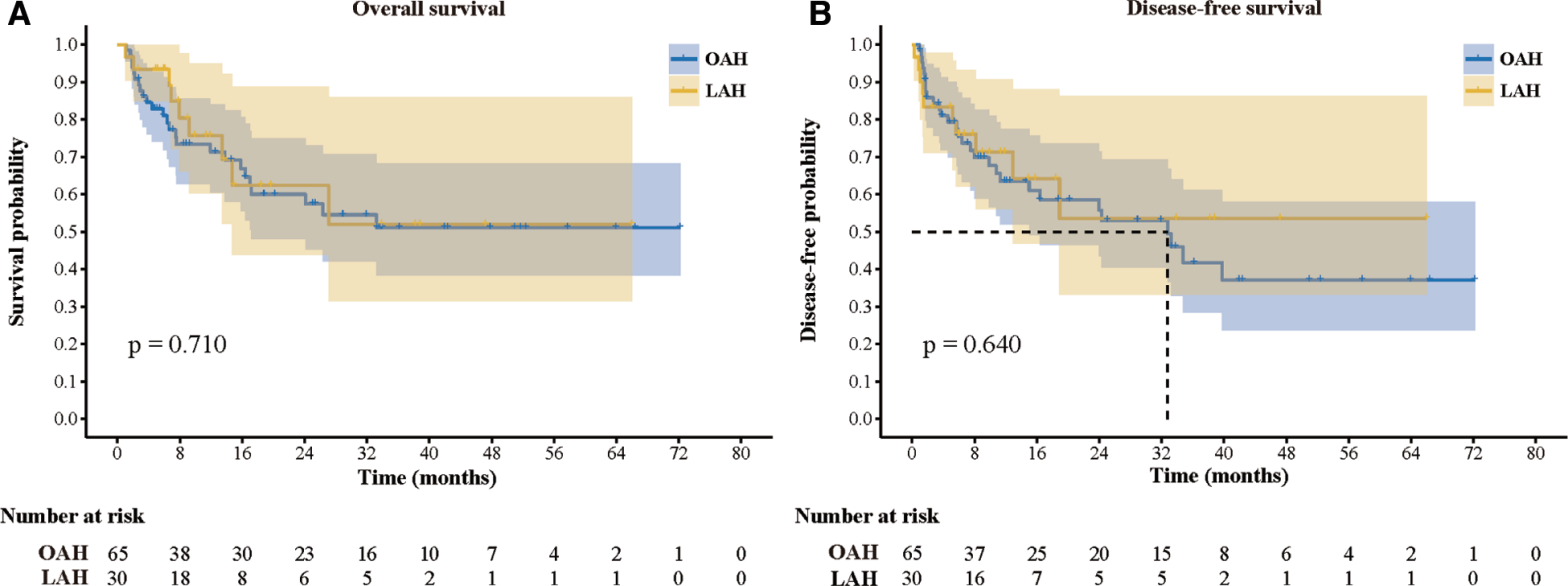
Comparison of overall survival and disease-free survival between the two groups. (**A**) Overall survival; (**B):** Disease-free survival.

A comparison of the follow-up and long-term outcomes between the OAH and LAH groups is shown in Table 3 and Figure 1. Sixteen patients (24.6%) in the OAH group and seven patients (23.3%) in the LAH group received subsequent therapy, with no statistically significant difference between the two groups. In addition, the two groups had similar results in terms of the use of specific subsequent therapy, such as hepatic artery chemoembolization, radiofrequency ablation, targeted therapy, immunotherapy, reoperation, chemotherapy, and radiotherapy (*P* > 0.05). The median follow-up time after surgery was 13.8 (1.1, 72.2) months in the LAH group and 10.7 (1.0, 66.0) months in the OAH group (*P* = 0.731). During the follow-up period, there were 29 (44.6%) cases of recurrence and 25 (38.5) deaths in the OAH group and 10 (33.3%) cases of recurrence and 9 (30.0) deaths in the LAH group. Both the total disease recurrence rate and total mortality rate were comparable between the two groups (*P* > 0.05). The 1- and 3-year OS rates were 71.3% and 51.1% in the OAH group and 75.7% and 52.0% in the LAH group, respectively (Figure 3A). The 1- and 3-year DFS rates were 63.4% and 41.7% in the OAH group and 71.3% and 53.5% in the LAH group, respectively (Figure 3B). There was no significant difference in the OS or DFS rate between the OAH and LAH groups (*P* = 0.640 and *P* = 0.710, respectively, Figures 3A,B).

### Univariate and multivariate analyses of factors associated with OS and DFS

The results of the univariate and multivariate analyses of variables that affect OS and DFS are shown in Tables 4, 5, respectively. Multivariate analysis showed that CA-199, CEA, HGB, tumor diameter, and T stage were independent prognostic factors for OS. Of these, CA-199 (HR 1.002, 95% CI 1.001–1.003, *P* = 0.001), CEA (HR 1.009, 95% CI 1.003–1.016, *P* = 0.006), tumor diameter (HR 1.284 95% CI 1.045–1.576, *P* = 0.017), and T stage (HR 5.105, 95% CI 1.126–23.149, *P* = 0.035) were independent risk factors for OS, but HGB (HR 0.962, 95% CI 0.938–0.986, *P* = 0.002) was an independent protective factor. Additionally, multivariate analysis showed that an elevated CA-199 level (HR 1.001, 95% CI 1.000–1.002, *P* = 0.018) and T stage > T2 (HR 3.893, 95% CI 1.281–11.836, *P* = 0.017) were independent risk factors for shorter DFS.

**Table 4:** Univariate and multivariate analyses of factors associated with overall survival rates.

Variable	Univariable cox regression analysis			Multivariable cox regression analysis		
	HR	95%CI	*P* value	HR	95%CI	*P* value
Sex male (vs. female)	1.863	0.932–3.726	0.079	2.475	0.995–6.155	0.051
Age (years)	1.041	0.999–1.084	0.056	1.030	0.981–1.081	0.232
BMI (kg/m^2^)	0.932	0.852–1.021	0.130			
ASA score						
1	-	-	-			
2	1.246	0.378–4.110	0.717			
3	0.951	0.159–5.695	0.956			
Diabetes yes (vs. no)	0.555	0.133–2.317	0.419			
Hypertension yes (vs. no)	0.774	0.350–1.712	0.527			
Coronary heart disease yes (vs. no)	0.045	0.000–25.024	0.337			
History of smoking yes (vs. no)	1.151	0.550–2.408	0.709			
History of alcohol consumption yes (vs. no)	1.371	0.678–2.772	0.380			
Hepatitis B virus infection yes (vs. no)	1.278	0.529–3.089	0.585			
Intrahepatic biliary lithiasis yes (vs. no)	1.595	0.659–3.860	0.300			
CA-199 (U/ml)	1.002	1.001–1.003	**< 0** **.** **001**	1.002	1.001–1.003	**0**.**001**
AFP (U/ml)	1.005	0.998–1.012	0.164			
CEA (U/ml)	1.009	1.004–1.013	**0**.**000**	1.009	1.003–1.016	**0**.**006**
Neutrophil count (10^9/L)	1.252	1.085–1.444	**0**.**002**	1.117	0.903–1.381	0.310
Lymphocyte count (10^9/L)	0.88	0.595–1.300	0.520			
Platelet count (10^9/L)	1.002	0.998–1.006	0.371			
HGB (g/L)	0.976	0.956–0.996	**0**.**018**	0.962	0.938–0.986	**0**.**002**
ALT (U/L)	0.999	0.994–1.003	0.610			
AST (U/L)	1.007	1.000–1.014	**0**.**048**	1.004	0.995–1.012	0.397
TBIL (umol/L)	1.003	0.998–1.007	0.231			
ALB (g/L)	0.951	0.882–1.025	0.187			
Tumor diameter (cm)	1.189	1.047–1.351	**0**.**008**	1.284	1.045–1.576	**0**.**017**
Tumor number multiple (vs. single)	0.666	0.203–2.184	0.502			
Pathological differentiation						
Poorly differentiated	-	-	-			
Moderately differentiated	0.463	0.220–0.975	**0**.**043**	1.539	0.552–4.289	0.409
Well differentiated	0.207	0.026–1.628	0.135	1.909	0.163–22.309	0.606
TNM stage > II (vs. ≤ II)	2.396	1.214–4.726	**0**.**012**	0.147	0.019–1.148	0.067
T stage > T2 (vs. ≤T2)	2.281	1.136–4.581	**0**.**020**	5.105	1.126–23.149	**0**.**035**
Microscopic metastatic foci yes (vs. no)	3.356	1.422–7.919	**0**.**006**	0.950	0.296–3.047	0.931
Microscopic perineural invasion yes (vs. no)	1.733	0.797–3.770	0.166			
Microscopic microvascular invasion yes (vs. no)	1.996	0.893–4.462	0.092	2.662	0.742–9.552	0.133
Lymph node dissection yes (vs. no)	1.126	0.573–2.209	0.731			
Nodal status positive (vs. negative)	1.954	0.908–4.204	0.087	2.617	0.580–11.818	0.211
Lymph node yield						
0 nodes	-	-	-			
1–5 nodes	1.234	0.615–2.474	0.554			
≥6 nodes	0.676	0.156–2.937	0.602			
Surgical margin R1 (vs. R0)	2.539	0.770–8.375	0.126			
Subsequent therapy yes (vs. no)	0.955	0.432–2.111	0.910			
LAH (vs. OAH)	0.864	0.402–1.859	0.709			

BMI, body mass index; ASA, American Society of Anesthesiology; CA-199, cancer antigen 19-9; AFP, alpha fetoprotein; CEA, carcinoembryonic antigen; HGB, hemoglobin; ALT, alanine aminotransferase; AST, aspartate aminotransferase; TBIL, total bilirubin; ALB, albumin; LAH, laparoscopic anatomical hepatectomy; OAH, open anatomical hepatectomy. Bold indicates statistical significance.

**Table 5:** Univariate and multivariate analyses of factors associated with disease-free survival rates.

Variable	Univariable cox regression analysis			Multivariable cox regression analysis		
	HR	95%CI	*P* value	HR	95%CI	*P* value
Sex male (vs. female)	1.408	0.747–2.653	0.290			
Age (years)	1.001	0.968–1.037	0.933			
BMI (kg/m^2^)	1.008	0.96–1.059	0.738			
ASA score						
1	-	-	-			
2	1.080	0.380–3.068	0.885			
3	1.050	0.234–4.708	0.950			
Diabetes yes (vs. no)	0.199	0.027–1.455	0.112			
Hypertension yes (vs. no)	0.504	0.222–1.143	0.101			
Coronary heart disease yes (vs. no)	0.044	0.000–10.635	0.265			
History of smoking yes (vs. no)	0.816	0.387–1.720	0.593			
History of alcohol consumption yes (vs. no)	0.941	0.468–1.892	0.865			
Hepatitis B virus infection yes (vs. no)	1.621	0.745–3.529	0.223			
Intrahepatic biliary lithiasis yes (vs. no)	1.893	0.834–4.297	0.127			
CA-199 (U/ml)	1.001	1.000–1.002	**0** **.** **003**	1.001	1.000–1.002	**0**.**018**
AFP (U/ml)	1.003	0.997–1.010	0.317			
CEA (U/ml)	1.003	0.998–1.009	0.247			
Neutrophil count (10^9/L)	1.239	1.083–1.416	**0**.**002**	1.180	0.984–1.416	0.074
Lymphocyte count (10^9/L)	0.943	0.805–1.103	0.463			
Platelet count (10^9/L)	1.004	1.000–1.008	0.061	1.004	0.999–1.008	0.100
HGB (g/L)	0.986	0.966–1.006	0.178			
ALT (U/L)	1.000	0.997–1.003	0.809			
AST (U/L)	1.006	0.999–1.013	0.080	0.999	0.991–1.007	0.815
TBIL (umol/L)	1.002	0.998–1.007	0.269			
ALB (g/L)	0.962	0.897–1.031	0.273			
Tumor diameter (cm)	1.182	1.047–1.334	**0**.**007**	1.126	0.961–1.320	0.143
Tumor number multiple (vs. single)	0.669	0.237–1.886	0.447			
Pathological differentiation						
Poorly differentiated	-	-	-			
Moderately differentiated	0.599	0.289–1.240	0.167			
Well differentiated	0.495	0.107–2.291	0.368			
TNM stage >II (vs. ≤II)	2.878	1.521–5.446	**0**.**001**	0.753	0.235–2.411	0.633
T stage >T2 (vs. ≤T2)	3.581	1.851–6.925	**< 0**.**001**	3.893	1.281–11.836	**0**.**017**
Microscopic metastatic foci yes (vs. no)	1.538	0.587–4.028	0.381			
Microscopic perineural invasion yes (vs. no)	1.522	0.706–3.283	0.284			
Microscopic microvascular invasion yes (vs. no)	2.153	1.024–4.525	**0**.**043**	1.761	0.686–4.519	0.239
Lymph node dissection yes (vs. no)	1.083	0.578–2.031	0.803			
Nodal status positive (vs. negative)	1.605	0.758–3.400	0.216			
Lymph node yield						
0 nodes	-	-	-			
1–5 nodes	1.145	0.593–2.211	0.688			
≥6 nodes	0.842	0.249–2.841	0.781			
Surgical margin R1 (vs. R0)	1.598	0.383–6.667	0.520			
Subsequent therapy yes (vs. no)	1.546	0.793–3.015	0.201			
LAH (vs. OAH)	0.841	0.409–1.729	0.637			

BMI, body mass index; ASA, American Society of Anesthesiology; CA-199, cancer antigen 19-9; AFP, alpha fetoprotein; CEA, carcinoembryonic antigen; HGB, hemoglobin; ALT, alanine aminotransferase; AST, aspartate aminotransferase; TBIL, total bilirubin; ALB, albumin; LAH, laparoscopic anatomical hepatectomy; OAH, open anatomical hepatectomy. Bold indicates statistical significance.

## Discussion

In the last two decades, laparoscopic hepatectomy (LH) has progressed rapidly with the development of laparoscopic techniques and the advancement of laparoscopic instruments, and laparoscopic surgery has become feasible in some complex and difficult cases in which LH was previously considered difficult. In 2002, Chen first reported LH for ICC and successfully performed LND laparoscopically (11); since then, studies on LH for ICC have emerged. In most of these studies, laparoscopic surgery has been suggested to be associated with lower morbidity rates, less pain, faster recovery, and shorter hospital stays than conventional open surgery in terms of short-term outcomes (12–15). However, none of those studies have explored the advantages and disadvantages of the two approaches in terms of short-term outcomes after anatomical hepatectomy. The present study therefore aimed to fill this gap in knowledge and identified that the intraoperative blood loss, incision length, and length of postoperative hospital stay were more favorable in the LAH group than in the OAH group. Although these findings require confirmation in larger-scale trials, they are nevertheless encouraging and indicate that the advantages of minimally invasive techniques were retained despite anatomical hepatectomy increasing the technical difficulty of laparoscopic surgery. Moreover, LAH showed encouraging results in terms of the operative duration, despite this factor being reported as a disadvantage of laparoscopic surgery in previous studies (16). In our study, although the operative duration was slightly longer in the LAH group than the OAH group, the difference was not statistically significant. We believe that this is because the surgeons had already accumulated sufficient experience to overcome the learning curve of LH, as they performed a large number of LH surgeries at our center. In this study, the proportions of trisectionectomy and hemihepatectomy in the LAH group were significantly lower than those in the OAH group, while the proportions of central bisectionectomy, sectionectomy and segmentectomy in the LAH group were significantly higher than those in the OAH group. These findings suggest that LAH was likely to enable the resection of a lower volume of liver tissue, which was also found in previous studies (14, 17). We think that this phenomenon can be explained by the clear but not statistically significant difference in tumor diameter between the two groups in our study. The tumor diameter was much larger in the OAH group, which inevitably led to the need for more extensive resection. Nonetheless, this finding also indicates the possibility of patient selection bias, which is one of the limitations of this study. In terms of postoperative complications, Hobeika et al. studied 548 ICC patients who underwent laparoscopic and open surgery and found that the incidence of overall complications and severe complications was lower in the laparoscopic group than in the open group but that the difference was not significant; this trend has been observed in most of the previous studies (15, 16). Similarly, our study also found a downward trend in the incidence of complications in the LAH group, including grade 3 or 4 complications, incision-related complications, postoperative hemorrhage, and delayed gastric emptying, among others, but again, with no significant differences.

There is still controversy over the need for routine LND in patients with ICC. Some opponents argue that LND is not beneficial for ICC patients because LND fails to improve OS or DFS in such patients and instead leads to an increase in postoperative complications (15, 16, 18, 19). However, proponents argue that routine LND in ICC patients is beneficial, as they believe that LND not only prolongs OS and DFS but also allows for accurate lymph node staging, which can help in determining the patient's prognosis and developing subsequent adjuvant treatment plans (20, 21). Although there is a consensus among some current recommendations that routine LND should be performed in patients with ICC, there is still a gap between current clinical practice and these guidelines. Our study also points to another important issue: the LND rate was lower in the LAH group than in the OAH group. This is not a coincidental finding and has been mentioned in several previous studies. Hobika et al. found a lower probability of LND in the laparoscopic group than in the open group in a nationwide study (12). In addition, Martin et al. suggested that laparoscopic treatment for ICC was associated with worse lymph node evaluations than open surgery (22), while several other meta-analyses also concluded that the LND rate was lower in the laparoscopic group (23, 24). We believe that the main reasons for this are the high degree of technical difficulty in performing LND laparoscopically and the lack of a consensus on the use of routine LND in clinical practice, which leads to a preference for not performing LND when left to the discretion of the laparoscopic surgeon. Reassuringly, this divide seems to have improved in recent years with the advancement of laparoscopic techniques and the development of laparoscopic instruments. In a recent single-center study, Ratti et al. demonstrated that laparoscopic liver resection (LLR) can achieve a higher percentage of complete LND and fewer LND-related complications (25). Moreover, the da Vinci surgical platform, which has become more widely used in recent years, provides an expanded three-dimensional view and greater degrees of freedom through the articulating arms, and we have reason to believe that this tool will make LND even easier.

The lack of haptic feedback is one of the main disadvantages of laparoscopic surgery in clinical practice because it may preclude surgeons from accurately judging the location of certain portions of the tumor boundary. This can lead to an increased rate of positive surgical margins during the operation and inevitably result in a poorer prognosis. Theoretically, *en bloc* resection, avoiding a positive surgical margin and any residual tumor, reduces tumor growth and metastasis and therefore results in better OS and DFS; this view has been demonstrated in previous studies (26, 27). However, in the current study, we did not find any association between surgical margin and OS or DFS on either univariate or multivariate regression analysis, which we speculate may result from the bias related to the small sample size. Reassuringly, similar surgical margin outcomes were achieved in the two groups, indicating that LAH could reach the same oncologic adequacy as OAH. We suspect that this could be due to the surgeon's skill level and advantages of anatomical hepatectomy in achieving oncologic adequacy, which compensated for the deficiencies in haptic feedback. In the present study, similar OS and DFS rates were achieved in the LAH and OAH groups, which is consistent with the findings of most previous reports (14, 15, 23, 24). Additionally, both the total disease recurrence rate and total mortality rate were comparable between the two groups. This strongly suggests that compared with OAH, LAH can achieve similar long-term outcomes and is a safe and feasible alternative treatment for ICC patients.

This study has several limitations. First, this study was not a randomized controlled trial, so patient selection bias may be present. Second, this was a single-center study, and we further screened the sample to include patients undergoing anatomical resection, which resulted in a small sample size and further resulted in insufficient statistical power. Therefore, there is a need for future large-sample, multicenter, and high-quality interventional studies comparing LAH with OAH in ICC. Finally, the data on subsequent therapy, recurrence and mortality in this study relied on the retrospective recall of the patients or their families, which may have resulted in recall bias. Moreover, some parameters of the subsequent therapeutic strategies, such as number of cycles, regimen, dose, etc., were not listed; only whether the patient received some kind of subsequent therapy was recorded, thereby leading to a limited interpretation of each patient's prognostic outcome.

## Conclusion

In conclusion, LAH for ICC is safe and feasible when performed by experienced surgeons. Furthermore, our study revealed that LAH provides better short-term outcomes than OAH and leads to a comparable long-term prognosis.

## Data Availability

The raw data supporting the conclusions of this article will be made available by the authors, without undue reservation.
